# Computational Methods for Resting-State EEG of Patients With Disorders of Consciousness

**DOI:** 10.3389/fnins.2019.00807

**Published:** 2019-08-06

**Authors:** Silvia Corchs, Giovanni Chioma, Riccardo Dondi, Francesca Gasparini, Sara Manzoni, Urszula Markowska-Kaczmar, Giancarlo Mauri, Italo Zoppis, Angela Morreale

**Affiliations:** ^1^Department of Computer Science, University Milano-Bicocca, Milan, Italy; ^2^Behavioral Neurology, Montecatone Rehabilitation Institute, Imola, Italy; ^3^Department of Letter and Communication, University of Bergamo, Bergamo, Italy; ^4^Department of Computational Intelligence, Faculty of Computer Science and Management, Wrocław University of Science and Technology, Wroclaw, Poland

**Keywords:** computational methods, EEG, DOC, VS, MCS, machine learning, resting state analysis, deep learning

## Abstract

Patients who survive brain injuries may develop Disorders of Consciousness (DOC) such as Coma, Vegetative State (VS) or Minimally Conscious State (MCS). Unfortunately, the rate of misdiagnosis between VS and MCS due to clinical judgment is high. Therefore, diagnostic decision support systems aiming to correct any differentiation between VS and MCS are essential for the characterization of an adequate treatment and an effective prognosis. In recent decades, there has been a growing interest in the new EEG computational techniques. We have reviewed how resting-state EEG is computationally analyzed to support differential diagnosis between VS and MCS in view of applicability of these methods in clinical practice. The studies available so far have used different techniques and analyses; it is therefore hard to draw general conclusions. Studies using a discriminant analysis with a combination of various factors and reporting a cut-off are among the most interesting ones for a future clinical application.

## 1. Introduction

Research on Disorders of Consciousness (DOC) is currently an important challenge for physicians and neuro-scientists involved in differential diagnostics between Minimally Conscious State (MCS) and Vegetative State (VS). In fact, due to the variability of the patient's cognitive awareness, the rate of misdiagnosis between VS and MCS is still high, being currently estimated around 40%, although the use of a behavioral scale could ameliorate the diagnostic accuracy (Schnakers et al., 2009). Moreover, MCS patients generally show greater responses to treatments, and thus better prognosis results (Bai et al., 2017a). It follows that, the high risk of misdiagnosis affects both the neuro-rehabilitation planning, and the caregiver's roles and objectives (Estraneo et al., 2016a; Bai et al., 2017a).

The application of new neuroimaging techniques opens up new diagnostic possibilities (Gosseries et al., 2019; Jang et al., 2019; Tan et al., 2019). However, these methods are not always readily available even at dedicated neurorehabilitation centers. In this context electroencephalogram (EEG) is still one of the most popular approach for data acquisition on cerebral activity (Fingelkurts et al., 2013). Its high temporal resolution, low cost and safety make it effective for DOC to discriminate between VS and MCS patients (Bagnato et al., 2015; Bender et al., 2015; Bai et al., 2017a; Estraneo et al., 2017). In these cases, a resting-state EEG analysis is applied to evaluate the brain electrical activity in absence of tasks and instructions (Bai et al., 2017a). Other current EEG-related techniques in DOC are Event-Related Potential (ERP) (Signorino et al., 1995; Faugeras et al., 2011; Morlet and Fischer, 2014), Transcranial Magnetic Stimulation (TMS)-EEG (Casali et al., 2013) and EEG with transcranial direct current stimulation (tDCS) (Bai et al., 2017b). Furthermore, resting-state EEG, flanked by appropriate quantitative methods (i.e., QEEG), provides objective clinical assessment thus avoiding subjective errors (Stefan et al., 2018) in the clinical practice for diagnosis (Sitt et al., 2014), prognosis (Chennu et al., 2017), and treatment evaluation (Bai et al., 2017b, 2018; Estraneo et al., 2017; He et al., 2018).

In this paper, we assess the usefulness of QEEG analysis of resting-state activity, enhanced by adequate computational methods to disentangling VS from MCS patients. We review the current scientific literature, and focus on the methodological procedures applied for resting-state EEG pre-processing, inference and (machine) learning techniques. Our purpose is to magnify any possible applications of inferential analysis of EEG data to diagnostic question, and how this process could reduce errors correlated to clinician subjective assessment.

## 2. Methods

Many authors have adopted various approaches to analyze EEG features in DOC. By following the steps adopted for the EEG analysis, we survey the current literature using Medical Subject Headings (MSH) terms—Resting-state-EEG, DOC, VS, MCS, and diagnosis, associated with clinical and computational researches in pre-processing, feature extraction, and inferences. In particular, unlike other reviews on DOC research (e.g., Bai et al., 2017a), here the focus is on the following items.

1. Diagnosis: We focused on studies aiming to disentangle VS and MCS patients. Although the number of corresponding works are still relatively lacking, several authors have begun to document the application of effective features.
2. Computational methods: We surveyed structured approaches for (relational) learning, and inferential analysis. These studies are mainly delivered from machine learning communities and graph theory concepts. In this case, only a low number of studies oriented to our main diagnostic question (i.e., VS and MCS differentiation) were found. Therefore, we extended the research to the whole class of DOC literature.

Eventually, we excluded the studies on sleep recordings.

## 3. Results

To the best our knowledge, 17 articles have been published in which resting-state EEG analysis is used to disentangle the diagnosis of VS from MCS. Next sections report how these authors approach the analysis of raw resting-state EEG data in pre-processing, feature construction and inference. More information about these studies are summarized in Tables 1, 2.

**Table 1:** Studies characteristics and results.

**Articles**	**Sample**	**Etiology**	**Duration**	**Scale**	**Methods and features**	**Results**
Stefan et al. (2018)	51 VS, 11 MCS	14 T, 48 NT		CRS-R	FC, CM, MS, ApEn, PeEn, Coh, wSMI, STE	Duration of MS d alpha AUC 74%
Naro et al. (2018)	17 VS, 15 MCS	10 T, 22 NT	12,56 months	CRS-R	SA, FC, SPO, dWPLI	Delta, alpha, dWPLI *P* ≤ 0.05
Chennu et al. (2017)	23 VS, 66 MCS	51 T, 53 NT		CRS-R	FC, dWPLI, others seven measures	PC alpha 79% accuracy, SVM classifier 74% accuracy
Schorr et al. (2016)	58 VS, 15 MCS	18 T, 55 NT	585,1 days	CRS-R	SA, FC, SPO, Coh	No positive results
Estraneo et al. (2016b)	37 VS, 36 MCS	21 T, 52 NT	>3 months	CRS-R	SA, PB	Pattern LV sensibility 95%, specificity 38.95%
Piarulli et al. (2016)	6 VS, 6 MCS	7 T, 5 NT	69 days	CRS-R	SA, CM, SPO, SpE, WD	Alpha, beta1, theta, delta, SpE *P* ≤ 0.05, WD specific MCS component
Engemann et al. (2018)	21 VS, 57 MCS	37 T, 41 NT	1040,6 days	CRS-R	SA, FC, CM, 28 biomarkers	DOC Forest AUC 0.75
Höller et al. (2013)	27 VS, 22 MCS	13 T, 41 NT	10,33 months	CRS-R	FC, CM, 12 measures (mostly from biosig toolbox)	Partial Coh 0.96 accuracy
Fingelkurts et al. (2013)	14 VS, 7 MCS	9 T, 12 NT	57 days	LCF	SA, Spectral oscillation	*P* ≤ 0.05
Lechinger et al. (2013)	8 VS, 9 MCS	8 T, 9 NT	75,63 months	CRS-R	SA, SPO	No positive results
Lehembre et al. (2012)	10 VS, 18 MCS	13 T, 15 NT	<3 months	CRS-R	SA, FC,SPO, Coh, IC, PLI	SPO delta and alpha, IC and PLI front—post theta *P* ≤ 0.05
Fingelkurts et al. (2012)	14 VS, 7 MCS	9 T, 12 NT	<3 months	LCF	SA, Spectral oscillation	Alpha SP 26% VS, 37% MCS
Gosseries et al. (2011b)	24 VS, 26 MCS	23 T, 33 NT		CRS-R	CM,State entropy, Response entropy	Specificity and sensibility 77% (only acutes)
Wu et al. (2011a)	21 VS, 16 MCS		<6 months	GCS, RCC, CRS-R	SA, CM, SPO, LZC, ApEn, C-ApEn	No positive results
Wu et al. (2011b)	30 VS, 20 MCS	25 T, 25 NT	VS 112.2 MS 139,2 days	GCS, RCC	CM, ApEn, C-ApEn	C-ApEn *P* ≤ 0.05
Schnakers et al. (2008)	13 VS, 30 MCS	16 T, 27 NT		CRS-R, GCS	SA, BIS, three derived measures	BIS *P* ≤ 0.05
Khanmohammadi et al. (2018)	54 patients GCS <8			GCS	FC, INRI others	Groups based on GCS *P* ≤ 0.05

*VS, Vegetative State; MCS, Minimally Conscious State; T, Traumatic; NT, Non-traumatic; CRS-R, Coma Recovery Scale-revised; GCS, Glasgow Coma Scale; LCF, Level of Cognitive Functioning; RCC, Rappaport Coma/Near coma Scale; SA, Spectral analysis; FC, Functional connectivity; CM, Complexity measures; MS, Microstate analysis; LV, Low Voltage; ApEn, Approximate Entropy; C-ApEn, Cross-Approximate Entropy; PeEn, Permutation Entropy; SPO, Spectral Power; SpE, Spectral Entropy; Coh, Coherence; IC, Imaginary Coherence; STE, Symbolic Transfer Entropy; wSMI, weighted Symbolic Mutual Information; dWPLI, Debiased Weighted Phase Lag Index; CNA, Complex Network Analysis; PLI, Phase Lag Index; LZC, Lempel-Ziv Complexity; BIS, Bispectral. Index; PB, Predominant background activity; WD, Wavelet decomposition; INRI, Intrinsic Network Reactivity Index*.

**Table 2:** Acquisition and preprocessing.

**Articles**	**Number of electrodes**	**F_*C*_**	**Filtering**	**Artifact removal**	**Rythms**
Stefan et al. (2018)	HDE 256	1,000 Hz	0.1 Hz	Channel removal, statistic thresholding	Delta (0–4 Hz), theta (4–8 Hz), alpha (8–13Hz), 2–20 Hz
Naro et al. (2018)	32	512 Hz	0.1–45 Hz	Epoch removal, visual inspection and ICA	Delta (0–4 Hz), theta (4–8 Hz), alpha (8–13 Hz), Beta (14–29 Hz), gamma (25–40 Hz)
Chennu et al. (2017)	HDE (256)	500 Hz	0.5–45 Hz	N/A	Delta (0–4 Hz), theta (4–8 Hz), alpha (8–13 Hz)
Schorr et al. (2016)	HDE (256)	250 Hz	1–100 Hz, notch 50 Hz	Epoch removal, thresholding and visual inspection	Delta (1–4 Hz), theta (5–8 Hz), alpha (9–13 Hz), Beta (14–30Hz), gamma (30–100 Hz)
Estraneo et al. (2016b)	19	N/A	1–70 Hz, notch	Synchronous video to remove artifacts due to subjects' movements	Delta (0–4 Hz), theta (4–8 Hz), alpha (8–13 Hz)
Piarulli et al. (2016)	12	500 Hz	1–45 Hz	Epoch removal, thresholding on EMG and EOG and visual inspection	Delta (1–3.75 Hz), theta (4–7.75 Hz), alpha (8–11.75 Hz), beta1 (12–17.75 Hz), beta2 (18–24.75 Hz)
Engemann et al. (2018)	256	250 Hz	0.2–45 Hz	Thresholding	Delta (1–4 Hz), theta (4–8 Hz), Alpha (8–13 Hz)
Höller et al. (2013)	32	1,000 Hz	1–48 Hz	ICA and visual inspection	–
Fingelkurts et al. (2013)	21	200 Hz	1–30 Hz	Visual inspection	Delta (1–2.5 Hz), theta1 (3–4 Hz), theta2 (4.5–5.5 Hz), theta3 (6–7 Hz), alpha1 (7.5–8.5 Hz), alpha2 (9–13 Hz)
Lechinger et al. (2013)	19	1,000 Hz	1–40 Hz	ICA and visual inspection	Delta (2–4 Hz), theta (4–8 Hz), alpha (8–12 Hz), Beta (12–30 Hz), gamma (30–40 Hz)
Lehembre et al. (2012)	10	500 Hz	0.5–48 Hz	Visual inspection	Delta (0.5–4 Hz), theta (4–8 Hz), alpha (8–12 Hz)
Fingelkurts et al. (2012)	21	500 Hz	1–30 Hz	Epoch removal, visual inspection	–
Gosseries et al. (2011b)	3	400 Hz	0.8–47 Hz	N/A	–
Wu et al. (2011a)	16	500 Hz	0.3–100 Hz	Visual inspection	–
Wu et al. (2011b)	16	500 Hz	0.3–100 Hz	Visual inspection	–
Schnakers et al. (2008)	N/A	256 Hz	0.3–70 Hz	Thresholding and visual inspection	–
Khanmohammadi et al. (2018)	19	500 Hz	1–45 Hz	–	–

### 3.1. Pre-processing

We did not find any common approach into the pre-processing phase of resting state EEG data, neither a common way to report the information about the followed steps. In the majority of the studies, EEG raw data were visually inspected by expert physicians. Most of the authors applied software libraries or environment (e.g., Brain Vision Analyzer, Matlab FASST toolbox, or Matlab fieldtrip toolbox) to target and easily identify potential noisy pieces of data and artifacts. In two studies (Chennu et al., 2017; Naro et al., 2018) authors applied Independent Component Analysis (ICA) algorithms, in one case (Gosseries et al., 2011a) procedures were fully automated with a particular device used for monitoring the level of the general anesthesia through entropy analysis. In Table 2 the pre-processing applied to raw data and the strategies adopted to remove noise are reported for each of the 17 considered articles.

### 3.2. Computational Methods for Feature and Inference Analysis

Relevant feature-based approaches are grouped here into three different categories, focused on *Spectral Power Analysis, Functional connectivity*, and *Complexity Measures*. A summary of the considered studies is reported in Table 1, while in Table 2 information about sampling frequency, number of electrodes, filtering and noise removal approaches are summarized. Further structured approaches, based on both inference and machine learning techniques are grouped into two categories: *Complex Network Analysis* and *Approaches for Inference and Learning*.

#### 3.2.1. Spectral Power Analysis

Spectral analysis is a well-established method for the analysis of EEG signals. The spectral profile (power spectrum) reflects the “frequency content” of the signal or, in other terms, the distribution of signal power over the frequency values. Several parameters derived from the spectrum have been applied for EEG quantification, including total power, spectral band power, and median and spectral edge frequency.

Well-known relationships between specific spectral power frequencies and awareness (i.e., alpha, delta and theta frequencies), are useful for supporting the diagnosis of DOC patients. For example, this method with its numerous variations showed that patients with DOC exhibit reduced power in the alpha range and increased power in the delta and theta range, with a more consistent difference presented in VS than MCS patients (Bai et al., 2017a; Stefan et al., 2018). In Table 2 rhythms and frequency bands studied by the analyzed bibliography are reported together with the results obtained.

#### 3.2.2. Functional Connectivity

Analysis of functional connectivity is the study of the synchronization of EEG *rhythms* in different areas of the brain observing specific EEG electrode groups in regions of interest (Varotto et al., 2014). From a statistical point of view, functional connectivity can be estimated by measuring the dependence among time series usually evaluated in terms of correlations or mutual information. In literature, there are many variants of such (dependency) estimation, which are in general defined to reveal in DOC patients, spatial links and potential disconnections related to the clinical state of the cases (Bai et al., 2017a; Stefan et al., 2018). Different functional connectivity indices can clearly give different results, as they are based on different underlying mathematical assumptions. It may therefore be difficult to select the most suitable method for identifying the appropriate strength of association. There are two main approaches to apply functional connectivity. One is generally known as “undirected” which infers whether two brain regions A and B are communicating in some general fashion, as typically revealed by the Pearson's correlation computed between their activity time series. For DOC patients specific coherence–based measures have been also applied (phase locking index, partial directed coherence, dWPLI, wSMI, imaginary part of coherence). On the other hand, “directed” functional connectivity (or “effective” connectivity) methods clarify asymmetries in activity flow that determine whether region A is communicating downstream to region B (or, respectively, B is communicating downstream to region A). In this case, indices, such as transfer entropy, symbolic transfer entropy, mutual information, and Granger casuality have similarly been applied to evaluate the corresponding signals information content.

#### 3.2.3. Complexity Measures

Mainly based on the definition of entropy, “complexity measures” characterize all those approaches used to evaluate the amount of information recorded by the system and represented, in this case, through the recorded EEG traces.

The main reason of the application of entropy-related techniques is based on the decreased complexity of EEG data in less aware patients. Several authors evaluated the entropy feature, like for example Gosseries et al. (2011a) who found that mean EEG entropy values were higher in MCS than in VS patients. These measure-based approaches can be applied in the time domain, such as approximate entropy, permutation entropy, Lempel-Ziv entropy, Kolmogorov-Chaitin complexity, and in the spectral power domain like spectral entropy. Permutation entropy and Kolmogorov-Chaitin complexity seems to be the most efficient techniques among the previous ones (Bai et al., 2017a).

#### 3.2.4. Complex Network Analysis

A particular way to study the structural–functional connectivity of the brain can be based on applying main graph-theoretical concepts and methodologies. Following this idea, a “Brain Network” is most commonly modeled using the definition of graph, which represents relationships between entities (i.e., objects broadly referred by an abstract set *V*), through a set of edges (i.e., abstract set of entities *E* representing ties between objects in *V*). Given a graph, represented as a pair *G* = (*V, E*), many typical problems, such as finding cohesive structures of vertexes which are connected in specific way (Dondi et al., 2016, 2017, 2018), or identifying the length of paths between entities, constitute fundamental issues which have been applied in several contexts. In these analysis, network connectivity can be easily created by thresholding measures of “association” between entities, e.g., in our cases electrodes, such that links in *E* are said to exist between two electrodes (vertexes in *V*) whether the corresponding correlation between the two electrodes exceed a certain threshold (Stefan et al., 2018).

Complex networks applied to EEG analysis for DOC patients constitute an emerging field which has already provided effective results (Chennu et al., 2017). Toppi et al. (2017) assessed patterns of connectivity and used graph theory to extract EEG indices describing the topology of resting state networks in DOC. They found that the main differences between VS/UWS and MCS patients can be discriminated by 2 classes of indices: (i) those describing the relationship between anterior and posterior areas of the brain, and (ii) those describing global properties of resting state networks, such as efficiency and tendency to create clusters.

#### 3.2.5. Approaches for Inferences and Learning

One of the early review article (Noirhomme et al., 2015) shows limitations and risks connected with using machine learning methods. Authors discuss the problem in the context of measurement paradigm, diagnostic protocol, limitation of patients and data analysis. In particular, attention was payed on the fact that the number of patients in the reviewed studies was relatively small. All studies relied on a binary classification between healthy controls and patients or between MCS and UWS patients. Multi-class classification was not reported in any study.

The most typical and probably mentioned (kernel-based) algorithm for classification is known as *Support Vector Machine* (SVM) (Boser et al., 1992; Vapnik, 1995, 1998). An example of its application for DOC analysis can be found in Engemann et al. (2015), Chennu et al. (2017), and Kafashan et al. (2017).Different approaches are given in Wielek et al. (2018) where standard scoring rules developed by American Academy of Sleep Medicine were applied. This model was evaluated on the basis of long-term EEG of DOC patients by using two machine learning methods: (i) a cluster analysis for a group-wise analysis with the aim of testing for presence of sleep-like clusters and (ii) a supervised classification. As an input for the classifier they used permutation entropy index whose robustness against environmental noise renders it more suitable for DOC analyses as compared to features based on the frequency spectrum. Moreover, they tested two classifiers (random forest vs. feedforward neural networks) observing finally that random forest provided slightly better results than neural networks.

Agglomerative hierarchical clustering is a bottom-up method producing a tree of clusters (called a dendrogram) whose hierarchy depends on the degree of similarity between observations represented by n-dimensional feature vectors. It was applied to check whether similar sleep-related patterns exist across groups (i.e., healthy, MCS, and VS patients). Engemann et al. (2015) describe a system that extracts statistically validated EEG-measures quantifying biomarkers of consciousness and statistical model that predict an incoming patient's state of consciousness. Their study is focused on evaluation of predictive power of various EEG measures. SVM was used as a classifier.

## 4. Prospective Directions

Deep learning is now used intensively in various domains, and is receiving promising results in solving many problems. Two kinds of networks are widely used—Convolutional Networks (CNN) (LeCun and Bengio, 1998) and Recurrent Networks which is dedicated to sequences processing. Currently, recurrent neural networks are mainly based on Long Short-Term Memory (LSTM) (Sepp Hochreiter and Schmidhuber, 1997) or Gated Recurrent Unit (GRU) networks (Cho et al., 2014), that include gates preventing vanishing or exploding gradient.

There are not many papers describing an application of deep networks in DOC domain, probably because of a minimal number of patients and EEG samples in comparison to the need of deep networks training. The most relevant paper has been already cited Craig et al. (2018) use Deep Graph Convolutional Neural Networks.

In our opinion, it is worth noticing some trials that show new research direction in EEG processing to build EEG measures. Schirrmeister et al. (2017) showed that convolutional network applied to EEG signal could reach accuracies at least in the same range as a *filter bank* common spatial pattern (FBCSP) for decoding task-related information from EEG. Authors designed the visualizations to show how Convolutional Nets use the amplitude of spectral band power features. One straightforward extension would be to apply these visualizations to show how Convolutional Nets use the amplitude of the raw time-domain EEG signal. This visualization could give insights into discriminative time-domain features, such as event-related potentials.

Zafar et al. (2017) proposed hybrid algorithms. They applied the Convolutional neural network with a t-test for the selection of significant features. Then, likelihood ratio-based score fusion was used for the prediction of brain activity. The proposed algorithm takes input data from multichannel EEG time series, which is also known as multivariate pattern analysis.

Bashivan et al. (2015) proposed recurrent convolutional network to preserve the spatial, spectral, and temporal structure of EEG which leads to finding features that are less sensitive to variations and distortions within each dimension. One of the challenges in modeling cognitive events from EEG data is finding representations that are invariant to inter- and intra-subject differences, as well as to inherent noise associated with EEG data collection. Authors propose a novel approach for learning such representations from multichannel EEG time-series, and demonstrate its advantages in the context of mental load classification task.

## 5. Conclusions

EEG signals carry valuable information regarding the brain system and it could also be used to provide different representations of brain's electrical activity that allow to define new computational problems, and to face with the discrimination between complex EEG signals.

As reported in this review, recent studies have shown that resting state EEG analysis is a useful tool to evaluate and disentangle VS from MCS patients. Some of these emerging ideas (e.g., based on graph theory) have already provided effective results. Therefore, in order to support clinicians in their daily diagnostic processes, it will be necessary to improve the efficiency of existing approaches by focusing on those translational researches aimed to apply these different relevant paradigms. Studies that used discriminant analysis, reporting a cut-off, with a combination of various factors are the most interesting for future clinical application.

## Author Contributions

IZ and AM conceived the study. SM and RD were involved in planning the work. AM and GC reviewed the literature for the clinical setting. IZ reviewed the literature for machine-learning based approaches. FG and SC reviewed the literature for preprocessing and feature construction approaches. RD and SM reviewed the literature for graph-based and AI-based approaches. UM-K reviewed the literature for deep learning approaches. UM-K and IZ reviewed the literature for perspective directions. GM, AM, and IZ supervised the work. All authors revised and approved the submitted version.

### Conflict of Interest Statement

The authors declare that the research was conducted in the absence of any commercial or financial relationships that could be construed as a potential conflict of interest.
