# Corrigendum: Computational Methods for Resting-State EEG of Patients With Disorders of Consciousness

**DOI:** 10.3389/fnins.2019.01323

**Published:** 2019-12-17

**Authors:** Silvia Corchs, Giovanni Chioma, Riccardo Dondi, Francesca Gasparini, Sara Manzoni, Urszula Markowska-Kaczmar, Giancarlo Mauri, Italo Zoppis, Angela Morreale

**Affiliations:** ^1^Department of Computer Science, University Milano-Bicocca, Milan, Italy; ^2^Behavioral Neurology, Montecatone Rehabilitation Institute, Imola, Italy; ^3^Department of Letter and Communication, University of Bergamo, Bergamo, Italy; ^4^Department of Computational Intelligence, Faculty of Computer Science and Management, Wrocław University of Science and Technology, Wroclaw, Poland

**Keywords:** computational methods, EEG, DOC, VS, MCS, machine learning, resting state analysis, deep learning

An author name was incorrectly spelled as **“Urszula Markowska-Kacznar.”** The correct spelling is **“Urszula Markowska-Kaczmar.”**

The authors apologize for this error and state that this does not change the scientific conclusions of the article in any way. The original article has been updated.

